# Virtual Screening Strategy to Identify Retinoic Acid-Related Orphan Receptor γt Modulators

**DOI:** 10.3390/molecules28083420

**Published:** 2023-04-13

**Authors:** Elmeri M. Jokinen, Miika Niemeläinen, Sami T. Kurkinen, Jukka V. Lehtonen, Sakari Lätti, Pekka A. Postila, Olli T. Pentikäinen, Sanna P. Niinivehmas

**Affiliations:** 1 MedChem.fi, Institute of Biomedicine, Integrative Physiology and Pharmacology, University of Turku, FI-20014 Turku, Finland; 2 InFLAMES Research Flagship Center, University of Turku, FI-20014 Turku, Finland; 3 Structural Bioinformatics Laboratory, Biochemistry, Faculty of Science and Engineering, Åbo Akademi University, FI-20500 Turku, Finland; 4 InFLAMES Research Flagship Center, Åbo Akademi University, FI-20500 Turku, Finland

**Keywords:** molecular docking, docking rescoring, negative image-based rescoring (R-NiB), brute force negative image-based optimization (BR-NiB), pharmacophore (PHA) filtering, retinoic acid receptor-related orphan receptor gamma t (RORγt), virtual screening (VS), inflammation

## Abstract

Molecular docking is a key method used in virtual screening (VS) campaigns to identify small-molecule ligands for drug discovery targets. While docking provides a tangible way to understand and predict the protein-ligand complex formation, the docking algorithms are often unable to separate active ligands from inactive molecules in practical VS usage. Here, a novel docking and shape-focused pharmacophore VS protocol is demonstrated for facilitating effective hit discovery using retinoic acid receptor-related orphan receptor gamma t (RORγt) as a case study. RORγt is a prospective target for treating inflammatory diseases such as psoriasis and multiple sclerosis. First, a commercial molecular database was flexibly docked. Second, the alternative docking poses were rescored against the shape/electrostatic potential of negative image-based (NIB) models that mirror the target’s binding cavity. The compositions of the NIB models were optimized via iterative trimming and benchmarking using a greedy search-driven algorithm or brute force NIB optimization. Third, a pharmacophore point-based filtering was performed to focus the hit identification on the known RORγt activity hotspots. Fourth, free energy binding affinity evaluation was performed on the remaining molecules. Finally, twenty-eight compounds were selected for in vitro testing and eight compounds were determined to be low μM range RORγt inhibitors, thereby showing that the introduced VS protocol generated an effective hit rate of ~29%.

## 1. Introduction

Molecular docking has an established role in protein structure-based drug discovery. However, often default scoring functions cannot identify the bioactive binding poses and therefore cannot successfully identify the active ligands in virtual screening (VS). Unfortunately, this happens even when the docking sampling has worked out satisfactorily [1,2,3]. A straightforward way to overcome this persistent docking scoring problem would be to rescore the already generated docking poses using a fast and accurate scoring method.

Negative image-based rescoring (R-NiB) is a protein cavity-based docking rescoring methodology that ranks docked compounds based on their shape and the electrostatic potential (ESP) complementarity with the protein’s binding cavity [4]. For this purpose, mirror images of the protein’s binding cavity known as negative image-based (NIB) models are generated using the cavity detection and filling software PANTHER [5]. The cavity-based docking rescoring, which is performed with the ultrafast similarity comparison algorithm ShaEP [6], has been shown to improve the yields with several docking algorithms and on multiple drug targets [4,7,8,9]. The NIB models can also be used directly in rigid docking known as NIB screening in which the ab initio generated ligand 3D conformers are aligned using ShaEP against the NIB model [10,11]. The NIB models can even be augmented with actual ligand fragments. This hybrid approach facilitated effective hit identification for phosphodiesterase 10A in rigid docking-based VS [12]. 

Although the PANTHER-generated NIB models have worked well in both rigid docking and docking rescoring [4,7,10,11,12], the model fitness can be improved substantially by tinkering with cavity detection settings or even by removing a few cavity atoms manually. This trimming can be done effectively via an automatic greedy search-driven method known as brute force negative image-based optimization (BR-NiB) [13]. In BR-NiB, the cavity atoms of the input NIB model are removed one at a time iteratively and the pool of new model variants (−1 cavity atom) are benchmark tested with ShaEP using a training set composed of known active ligands and inactive or decoy compounds. A large set of structurally diverse inactive or decoy compounds is essential for the optimization of a NIB model. Decoy compounds are used to validate the model’s performance in a realistic drug screening setting where few active molecules must be identified from among a large number of mostly inactive compounds [14]. The NIB model variant that generates the highest enrichment as estimated by ROCKER [15] at each generation is chosen for further editing and benchmarking until the enrichment improvement halts. Notably, the rescoring yield can be boosted yet more by incorporating protein-bound ligand(s) into the NIB models prior to the optimization in a process known as ligand-enhanced brute force negative image-based optimization (LBR-NiB) [16]. Because the model’s final composition is affected both by the improved shape/ESP scoring of active ligands and weakened ranking for the decoys, BR-NiB and LBR-NiB can be considered as shape-focused pharmacophore (PHA) modeling methods.

Modern drug discovery projects rely increasingly on cost-effective and target-tailored multi-method protocols that boost the identification of hit compounds in massive VS campaigns. Here, a novel docking-based VS protocol, relying on (1) flexible-ligand molecular docking, (2) cavity-based rescoring with BR-NiB-optimized NIB models, (3) point-based PHA filtering, and (4) molecular mechanics/generalized Born surface area (MM/GBSA) calculations, is pioneered and tested using retinoic acid receptor-related orphan receptor gamma (RORγ or NR1F3) as a case study. RORγ, which is a nuclear receptor acting as a transcription factor in the cytosol of various cells, has multiple roles in regulating circadian rhythm, metabolism, and immunity. An isoform of RORγ, RORγt or RORγ2, is expressed in various immune cells located mostly in the thymus where it has many roles in the regulation and activation of the immune system [17,18,19].

RORγt is important in mediating an immune response to aid in fighting against pathogens or destroying abnormal malignant cells in the body. However, constantly elevated expression of RORγt is connected to multiple inflammatory diseases such as arthritis, psoriasis, inflammatory respiratory diseases, autoimmune encephalomyelitis, multiple sclerosis, and inflammatory bowel disease [18,20,21]. Thus, RORγt is an appealing drug target for inflammatory conditions, and importantly, RORγt inhibition has already been demonstrated in vivo to reduce the symptoms of psoriasis-like skin inflammation, arthritis, and colitis in mice [22,23,24]; RORγt-deficient mice develop resistance to autoimmune encephalomyelitis and multiple sclerosis [25,26]. Lately, several clinical trials (Phase I/II) for treatment mainly of psoriasis with RORγt modulators have emerged, highlighting the potential and growing demand of effective RORγt inhibitors for therapeutic human use [27,28,29].

**Figure 1:**
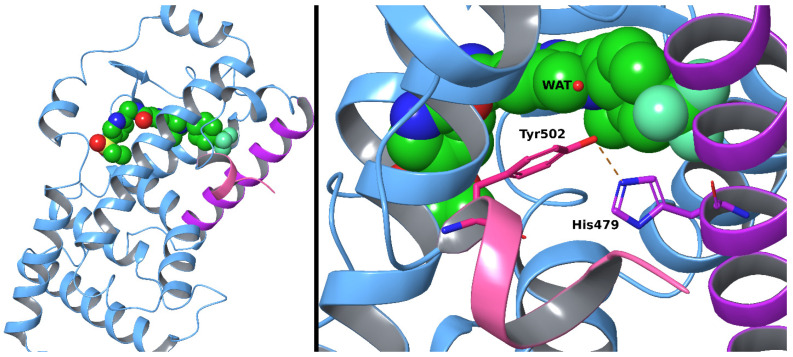
Retinoic acid receptor-related orphan receptor gamma t ligand binding domain. On the left is a 3D-structure of the ligand-binding domain of retinoic acid receptor-related orphan receptor gamma t (RORγt; PDB: 5NTW, cartoon representation) with a co-crystallized inverse agonist (CPK model with green carbons). On the right, the H-bond (orange dashed line) formed between His479 and Tyr502 (magenta and pink stick carbons, respectively) in helices 11 and 12 (magenta and pink cartoons, respectively) is shown. The atom coloring for O, N, S, and F is set to red, blue, yellow, and light green, respectively. Water (WAT) is shown as a red sphere.

RORγt-mediated inflammatory response initiates when an agonist is bound into the ligand-binding domain (LBD; Figure 1, left), stabilizing helix 11 positioning. This leads to a conformational change of the adjacent helix 12 that further stabilizes the allosteric binding site (ABS) and enables binding of a co-activator peptide. The activated RORγt shifts to the cell nucleus and binds as a monomer to the specific DNA sequences activating gene transcription responsible for inflammation. Antagonists block RORγt activation by preventing co-activator binding to the ABS. Inverse agonists stabilize ABS into a conformation that allows it to bind a co-repressor peptide instead of the co-activator. Co-repressor binding modulates the RORγt function in a manner that the physiological effects are the opposite of the agonist-bound RORγt. Consequently, the genes responsible for inflammation are downregulated, and anti-inflammatory genes are upregulated in the anti-inflammatory effect [18,27,30,31].

Modulation of the hydrogen bonding (H-bonding) between His479 in helix 11 and Tyr502 in helix 12 is crucial for determining whether the helix 12 conformation favors either co-activator or co-repressor binding [32]. Some inverse agonists facilitate co-repressor binding by preventing the inter-helix H-bond formation via steric obstruction. Alternatively, as a basis for inverse agonism, the movement of helix 12 has also been suggested to follow the release of a water molecule that is trapped into an unfavorably hydrophobic niche (Figure 1, right) [32].

Both structure-based and ligand-based VS approaches have been successfully employed to identify novel compounds for targeting RORγt in the literature [33,34,35,36,37,38,39]. Initial hits have demonstrated micromolar activities in the AlphaScreen assay and a cell-based reporter gene assay and, after optimization, a few compounds have displayed significantly enhanced RORγt inhibition with half maximal inhibitory concentration (IC_50_) values at the nanomolar level [35,36]. Previously, NIB screening was used to discover novel RORγt ligands, where pIC_50_ values ranged from 4.9 (11 μM) to 6.2 (590 nM) [37]. Molecular docking and/or molecular dynamics simulations have been successfully used to explore how agonist and inverse agonist compounds bind with the RORγt LBD [27,30,40,41]. Likewise, further optimization of absorption, distribution, metabolism, and excretion (ADME) and physicochemical properties with guidance from molecular simulations and modeling have assisted in improving compounds to achieve the level and duration of target engagement required for efficacy in the clinic [33].

Overall, these prior results provide a promising starting point for discovering new potent small-molecule RORγt inhibitors using the novel VS protocol pioneered in this study.

## 2. Results and Discussion

### Shape-Focused Pha Modeling and Docking Rescoring

**Figure 2:**
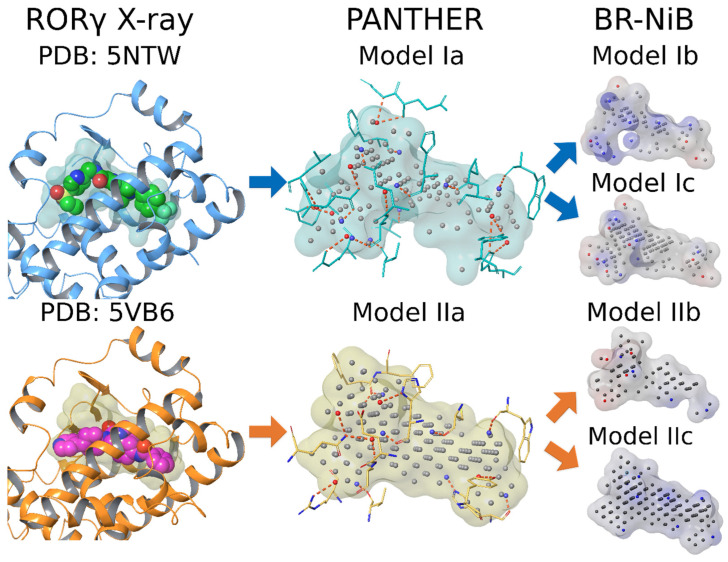
Negative image-based models of the RORγt binding cavity. The original cavity-filling negative image-based (NIB) models, Model Ia (PDB: 5NTW [32], cyan transparent surface) and Model IIa (PDB: 5VB6 [42]; yellow transparent surface), were trimmed with brute force negative image-based optimization (BR-NiB) using the complete set of active ligands from the ChEMBL database yielding Models Ib and IIb, respectively. Alternatively, the models were also optimized using only a subset of the ligands yielding to Models Ic and IIc (transparent surfaces with electrostatic potential). The protein 3D structures (blue/orange cartoon models) used in the NIB model generation with PANTHER are shown in complex with the co-crystallized ligands (CPK models with green/magenta carbons). The NIB models (transparent surfaces) are composed of neutral (C; gray), negatively charged (O; red), and positively charged (N; blue) cavity atoms. The projected H-bonds (orange dotted lines) of these cavity atoms with residues (cyan/orange stick models) lining the binding cavity are shown. Hydrogens are omitted for clarity.

Two NIB models of the RORγt binding cavity (Models Ia and IIa; Figure 2) were generated with PANTHER [5] and optimized using the BR-NiB method [13] before their docking rescoring usage (Figure 2). 

Out of the box, in the PANTHER-generated NIB models there is typically an excess of the cavity atoms that decrease their ability to find active ligands in docking rescoring usage (Figure 2). In BR-NiB, the effect of each cavity atom forming the NIB model, whether charged (N/O) or neutral (C), is subjected to systematic evaluation and editing in an automated greedy search [13]. The effect of each cavity atom or point in the NIB model is analyzed by removing them one by one and then testing the docking rescoring ability of each new model variant. This iterative process continues from generation to generation until the enrichment evaluation of the model does not show improvement. The numerical analysis of each model was done using Boltzmann-Enhanced Discrimination of Receiver Operating Characteristic with alpha value 20 (BEDROC20) [15,43] as the target metric. 

In theory, the optimized NIB models include key features from both the active ligands included in the training set and the inverted cavity itself. Moreover, the optimized NIB models should perform better in recognizing active ligands from inactive decoys than the non-optimized NIB models as suggested by the training set performance. However, because the training set composition affects the model optimization, both a full set and a subset containing only the lowest-ranked active RORγt compounds from the ChEMBL database [44,45,46] were used in the NIB model optimization. This dual approach was done to potentially avoid limiting the ability of the optimized models to recognize chemically diverse RORγt inhibitors in docking-based VS. 

**Figure 3:**
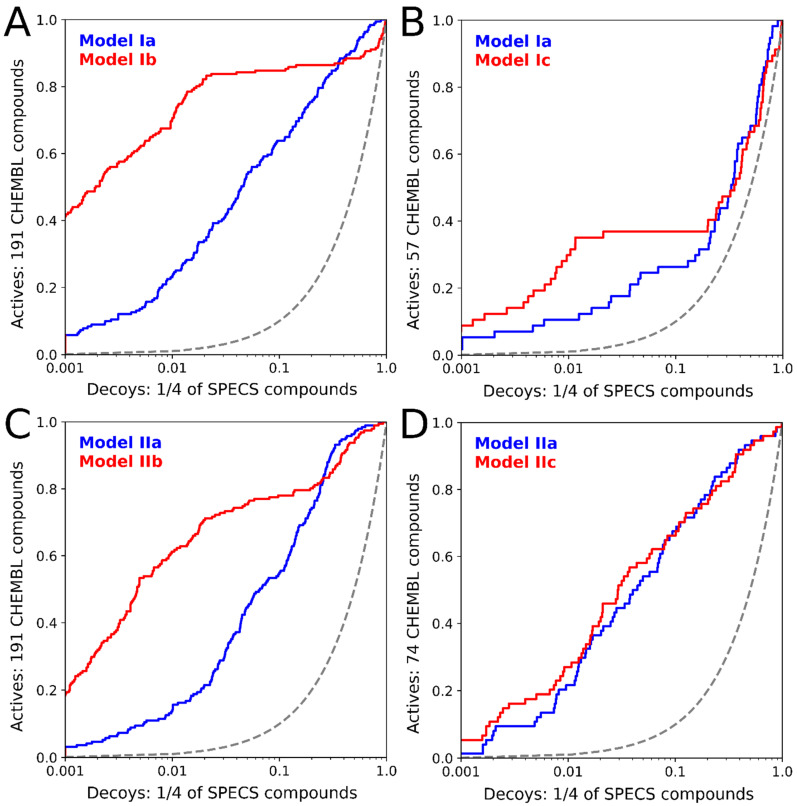
BR-NiB optimization of the original NIB models improved the identification of the active compounds. The semilogarithmic receiver operating characteristics curves show the following enrichments: PDB:5NTW models Ia and Ib with the complete set of active compounds (**A**), PDB:5NTW models Ia and Ic with the lowest-ranked active compounds (**B**), PDB:5VB6 models IIa and IIb with the complete set of active compounds (**C**), and PDB:5VB6 models IIa and IIc with the lowest-ranked active compounds (**D**). The blue graphs present the enrichment of the active compounds based on the original NIB models, while the red graphs present the enrichment based on the BR-NiB-optimized models. True positives are presented in the Y-axis, while false positives are shown in the X-axis [15,47]. The grey dotted line represents random classification of the compounds.

The BR-NiB processing significantly improved the effectiveness of the NIB models in docking rescoring usage (Figure 3). The BR-NiB-optimized models, describing the optimal shape/ESP features for filtering active RORγt ligands, were used to rescore the flexible docking poses of SPECS compounds. In practical terms, the docked SPECS compounds play a dual role in this study, first, they act as the decoy molecules in the NIB model optimization, and, second, the best ranked compounds were considered VS hits. 

## 3. Pharmacophore Filtering Focuses Compound Selection to Inverse Agonists

**Figure 4:**
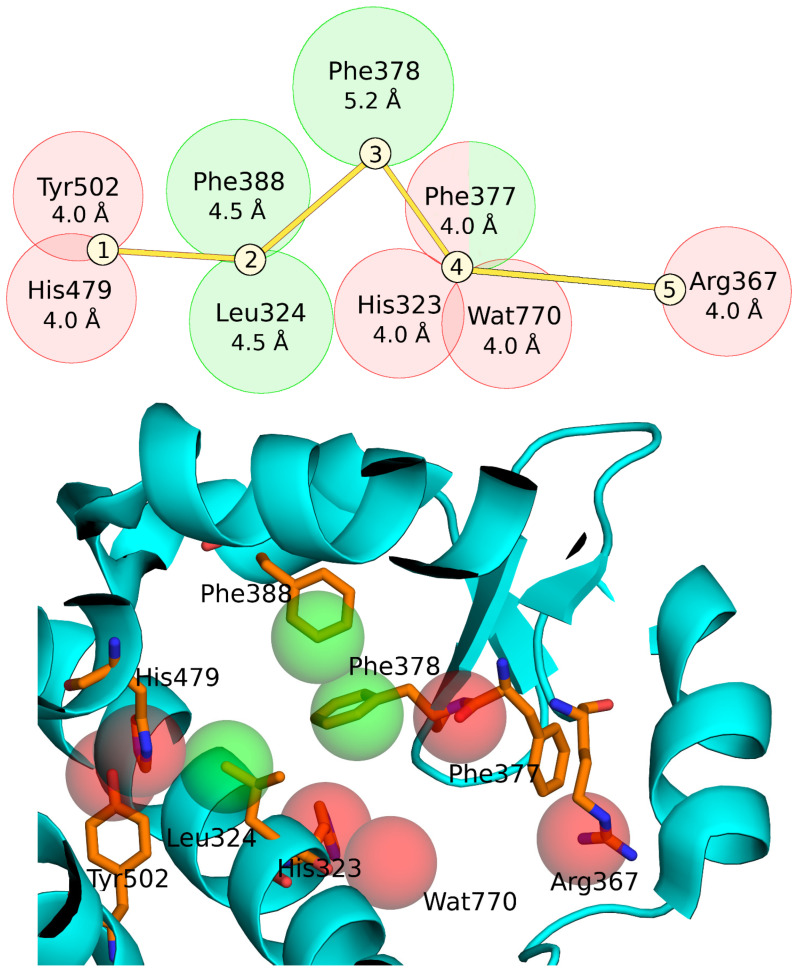
Pharmacophore point model used in SDFCONF filtering. On the top, 2D representation of the pharmacophore point model. Five important binding regions in the LBD of RORγt were identified. Two different combinations of the criteria were used in the final SDFCONF screening. Red circles demonstrate radiuses for hydrophilic groups and green circles radiuses for hydrophobic groups in respect to the selected residues. Below, the pharmacophore points depicted in the RORγt binding cavity (PDB: 5NTW). The protein is shown in cyan cartoon representation, important residues are shown in sticks with orange C, blue N, and red O atoms. The spheres indicating the positions of the pharmacophore points are colored as in the top figure. Note that the sphere sizes are not scaled to represent the used atom selection radii.

Five important binding regions in the LBD of RORγt were identified (Figure 4) based on a literature search [30,32] and visualization of available ligand-bound RORγt X-ray crystal structures. 

The first two regions are important for the stabilization of inverse agonist-binding conformation: (1) Two PHA points were defined at sites occupied by His479 and Tyr502, which can act as either H-bond acceptors or donors, and accordingly, non-hydrogen atoms (N, O, S, F, Cl, Br, or I) were sought; (2) Leu324 and Phe388 are important for forming hydrophobic or stacking effects with the binding ligand [30], and therefore, compounds not possessing any aromatic atom within 4.5 Å of Leu324 or Phe388 were excluded. Similarly, the third region (3) included Phe378 and compounds not possessing any aromatic atom within 5.2 Å of the residue were excluded. The fourth region (4) included His323 and the structural water molecule (Wat770), which can be either H-bond acceptors or donors [32,48]. Hence, the PHA filter points to exclude the compounds that did not show a possible H-bonding atom (simplified as N, O, S, F, Cl, Br, or I) within 4.0 Å of His323 or Wat770 were generated. Additionally, hydrophobic Phe377 sterically limits the fourth region. The fifth region (5) included Arg367, which acts as an H-bond donor. Therefore, a PHA filter point requiring an H-bond acceptor (demonstrated as heavy atoms N, O, S, F, Cl, Br, or I) was used. 

The PHA filtering, performed with SDFCONF [49], was fine-tuned by adjusting the optimal radii for the PHA points, which would discard as many decoy compounds as possible with a relatively low number of active compounds being discarded. Eventually, PHA filtering radii were determined to be 4.0 Å for region 1, 4.5 Å for region 2, 5.2 Å for region 3, 4.0 Å for region 4, and 4.0 Å for region 5. With these optimized settings (Figure 4), the PHA filtering excluded 83% of the SPECS compounds (with unknown activity) while only 37% of known active compounds were discarded. The discarded active compounds were mostly of low activity, suggesting that the combined BR-NiB and PHA filtering could assist in discovering compounds with a RORγt inverse agonism profile. 

## 4. Selecting Compounds for Experimental Testing

**Figure 5:**
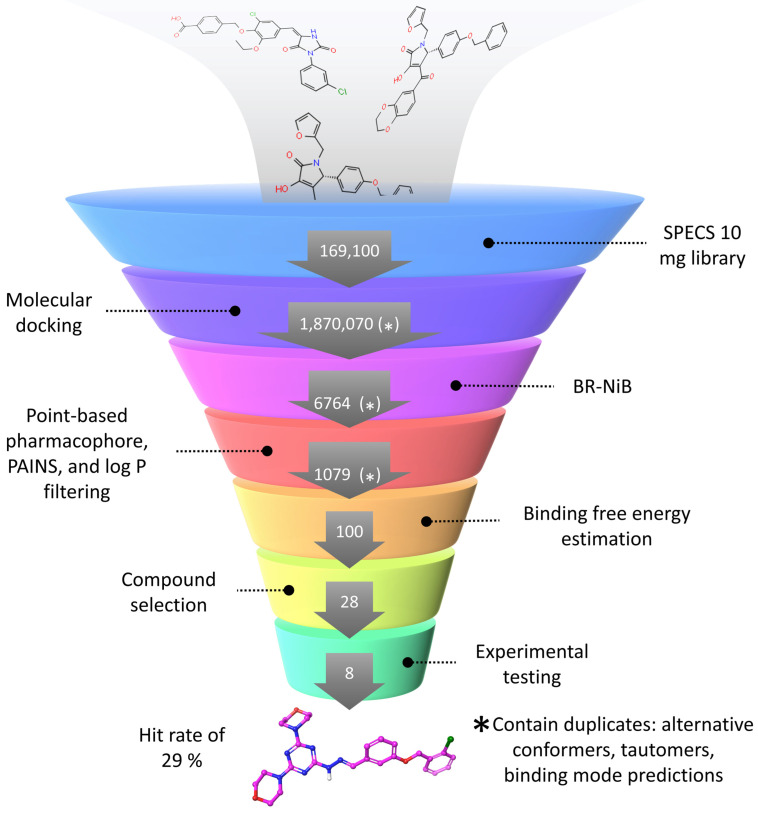
The docking and shape-focused filtering protocol used in virtual screening. (1) The SPECS 10 mg compound library set, which is composed of one hundred sixty-nine thousand and one hundred compounds, was flexibly docked and generated one million eight hundred seventy thousand and seventy entries for both protein structures. This number includes alternative tautomers, enantiomers, and ten docking poses for each compound referred here as duplicates (*). (2) After BR-NiB processing, one thousand six hundred and ninety-one top-ranked SPECS compounds were extracted for each optimized model (4 × 1691), yielding six thousand seven hundred and sixty-four entries. This number again includes duplicates. (3) Point-based pharmacophore and PAINS filtering was performed for the remaining compounds and, furthermore, logP filtering was done for the remaining compounds, yielding one thousand and seventy-nine entries with duplicates. (4) Next, one hundred top-ranked compounds were obtained based on the binding free energy calculations for all remaining compounds. (5) Twenty-eight compounds were selected for purchase after visual inspection. (6) Eight of the in vitro tested compounds were determined active at the low-micromolar range, yielding a hit rate of 29%.

First, the top 1% of the best-ranked SPECS compounds from BR-NiB (Models Ib, c and IIb, c; Figure 2) were selected for further consideration. Next, the molecules were screened with the PHA points defined from the RORγt ligand binding site using SDFCONF [49] (Figure 4). Third, the compounds were filtered by logP (< 5.5), and to avoid unspecific binding, the potential PAINS compounds were filtered out using Canvas. Fourth, Gibb’s free energy of binding was estimated with MM/GBSA calculations for the remaining compounds, discarding compounds with predicted binding energy > −95 kcal/mol. Finally, the binding complexes were evaluated visually to choose the most promising molecules for in vitro testing. In the manual selection of the compounds, special attention was paid to the diversity of their scaffold or core structures. Favorable steric packing at the binding cavity, as well as the number and quality of the formed interactions, were regarded to be vital. In particular, the disruption of the H-bond formation between His479 and Tyr502 was considered important, as this is favorable for inverse agonism [30]. Altogether, twenty-eight compounds were selected for experimental testing (Figure 5).

## 5. Novel Virtual Screening Strategy Yielded an Effective Hit Rate of 29%

**Figure 6:**
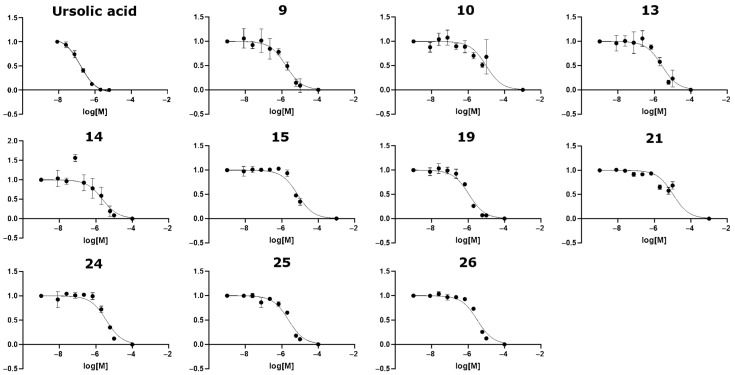
IC_50_ plots for the RORγt inverse agonists tested in this study. The IC_50_ plots are shown for the active compounds: **9**, **10**, **13**–**15**, **19**, **21**, **24**–**26**, and the control ursolic acid. For ursolic acid, the plot was formed by an average of three duplicate repeat measurements. A fourth duplicate measurement was omitted due to high measurement error at several ursolic acid concentrations.

**Figure 7:**
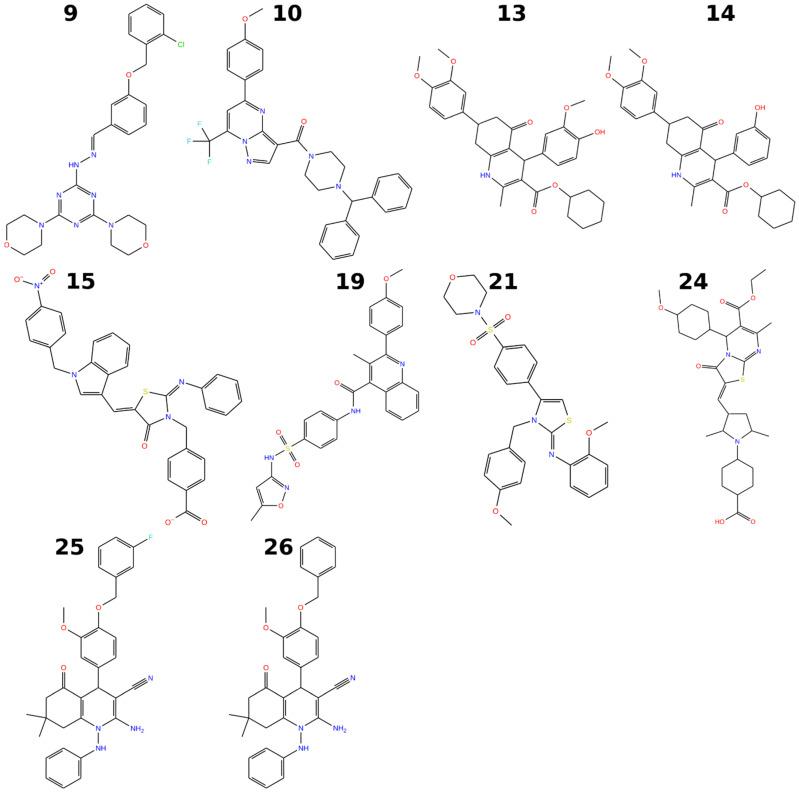
2D figures for the RORγt inverse agonists tested in this study. Figures are shown for the active compounds: **9**, **10**, **13**–**15**, **19**, **21**, and **24**–**26**.

The full RORγt inverse agonists appear to provide the most significant anti-inflammatory effect [27,28,29], and therefore, the discovery focused on finding potent novel full RORγt inverse agonists. A total of ten compounds (**9**, **10**, **13**–**15**, **19**, **21**, and **24**–**26**; Figure 6 and Figure 7) were identified to inhibit RORγt at some level. The estimated IC_50_ values ranged from 1.1 µM to 10.7 µM (Table 1; Figure 6). Compounds **19** and **9** were the most potent inverse agonists of RORγt with an IC_50_ of 1.1 µM and 1.7 µM, respectively (Table 1). For compounds **10** and **21**, the reported IC_50_ values should be considered only rough estimates, as the minimum signal intensity was not properly reached within the measured concentration range. Compounds **2** and **17** were non-soluble despite their predicted logP values indicating sufficient solubility (1.69 and 3.22 for compound **2** and 2.93 and 4.75 for compound **17**, as reported by the vendor or predicted by QikProp, respectively). The IC_50_ value of ursolic acid (positive control) was 185 nM, which was determined as an average of four duplicate measurements. Omitting one duplicate measurement with a high error provided an IC_50_ value of 157 nM, which is similar to previously reported values (Figure 6) [36,37].

Structural similarities between the active hit compounds and previously known RORγt inverse agonists or antagonists were studied by 2D molecular fingerprint comparisons (Table 1). Compound **24** was the only compound with a maximum Tanimoto coefficient > 0.2 due to the (7-methyl-3-oxo-5*H*-[1,3] thiazolo[3,2-a] pyrimidin-2(3*H*)-ylidene)methyl core that was also present within known ligands. Compound **19** contains a 4-(sulfonyl-phenyl)-quinolinecarboxamide, which was also detected in the set of known ligands, resulting in a slightly elevated maximum Tanimoto coefficient (0.171) for the most active VS hit. No close matches were found for compound **9**, which was the second most potent VS hit, although the 3-[(2-chlorobenzyl)oxy] benzaldehyde moiety of **9** had structural analogs among known ligands. Overall, most novel hit compounds had low Tanimoto coefficients (<0.15) compared to known RORγt ligands, indicating low structural similarity with previously known RORγt modulators.

Physicochemical and ADMET properties were calculated for the eight compounds that had well-defined experimental IC_50_ values (Table 2). Generally, the active hit compounds displayed drug-like properties as evaluated by the Lipinski’s rule of five [50]. All of the compounds had a single violation of the rule of five due to molecular weight moderately exceeding 500 Da. A second violation was caused by predicted logP > 5.0 for four compounds. For compounds **13** and **14**, QikProp predicted slightly higher logP values than Canvas. Compounds **9** and **19**, the two most active VS hits, contained no potentially reactive groups that could cause toxicity in vivo, and displayed sufficient properties related to human oral absorption and cell permeability. The ADMET predictions together with the molecular similarity analysis encourages the use of these compounds as starting points for the design of structurally novel and potent RORγt inverse agonists.

Both structure-based and ligand-based VS approaches have been successfully employed to identify novel compounds for RORγt [33,34,35,36,37,38,39]. For example, we have screened RORγt using molecular docking and NIB screening [37]. Both VS techniques mostly suggested the same compounds for experimental testing, supporting the viability of the in silico methods in molecular discovery. In Rauhamäki et al. eleven of the thirty-four experimentally tested molecules were verified as RORγt inverse agonists, yielding an effective hit rate of 32% [37], matching the results of this study. One of the first studies to use VS to find novel RORγt modulators was performed by Damm-Ganamet et al. using homology models of the LBD of RORγt in VS, and RORα co-crystal structures in recognizing important interactions across various chemical series. Of the one thousand seven hundred and fifty-seven hits prioritized by the VS, sixty-five diverse hits were active in experimental testing, resulting in a hit rate of 4.1% [39]. Zhang et al. used molecular docking to identify novel RORγt compounds and demonstrated that thirteen of the twenty-four tested molecules showed inhibitory activity [36]. Similarly, Song et al. utilized molecular docking and 3D shape similarity searching and identified eight of the twenty-eight tested compounds as RORγt inverse agonists [35]. In both studies, a relatively high concentration of 50 μM was used in the AlphaScreen assay, and the activity limit was set to 50% [36] or 35% [35] inhibition. In the study by Tan et al. two of the fifteen compounds suggested by molecular docking had therapeutic effects as shown in experimental studies [34]. Lugar et al. used VS to identify a promising scaffold for a starting point for the design of potent RORγ inhibitors [33]. Recently, Wu et al. utilized a new type of network-based VS to study RORγt modulators. Seven of the seventy-two compounds were confirmed to be low micromolar (IC_50_ from 0.1 μM to 4.97 μM) RORγt inverse agonists by in vitro experiments, resulting in a hit rate of 9.7% [38]. These studies show that VS can be used both successfully and diversely to identify novel RORγt compounds. Furthermore, a comparison to other VS studies using RORγt as a target shows that our methodology yields excellent hit rates.

## 6. Possible Binding Modes of the Most Active Inverse Agonists 9 and 19

**Figure 8:**
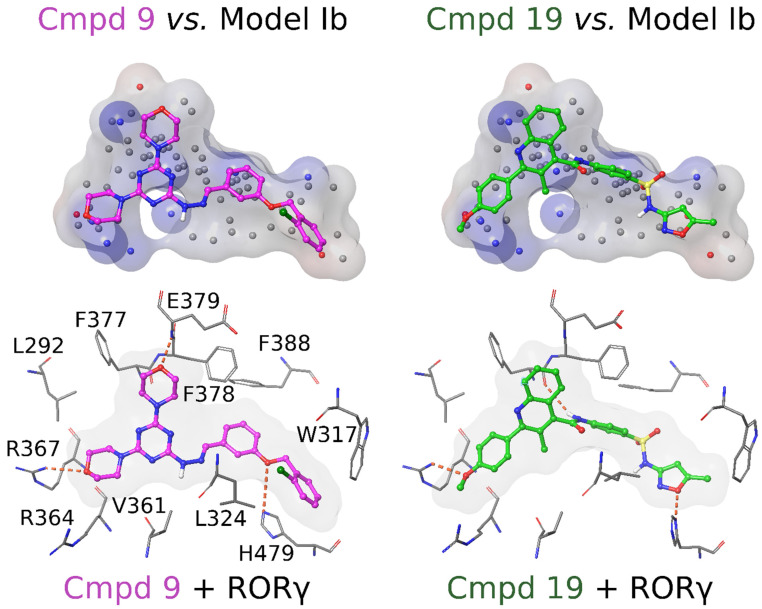
The proposed binding modes of compounds 9 and 19 with RORγt. On the top, both compounds **9** and **19** (ball-and-stick model with magenta/green carbons) show a high level of shape similarity with the BR-NiB-optimized Model Ib (transparent surface with electrostatic potential). Below, both of these compounds (gray shape-filling surface shown in the background) are also predicted to form H-bonds (orange dotted lines) with the key residues lining the RORγt binding cavity. For clarity, all hydrogens are omitted except for non-polar hydrogens of the compounds. See Figure 2 for further interpretation.

The predicted binding modes of the active compounds suggest many common features among them. Both compounds **9** and **19** bind across the entire cavity of the ligand-binding pocket (Figure 8). Morpholine groups of **9** accept H-bonds from Arg367 and Glu379. Similarly, a methoxy group of **19** accepts an H-bond from Arg367 and in the middle of **19** the amide group donates an H-bond to the main chain carbonyl oxygen of Phe377. Likewise, both form an H-bond with His479; in **9** an ether group accepts an H-bond from the epsilon position nitrogen of His479 and in **19** an isoxazole accepts an H-bond from the delta position nitrogen of His479. Thus, the length of the whole cavity is covered by H-bonds that stabilize the compounds to their places.

There are several favorable hydrophobic interactions between the amino acids lining the binding cavity and the active compounds. The two aromatic ring systems of **9** are packed against several residues: Trp317, Cys320, Leu324, Met365, Phe378, Phe388, Leu391, Leu396, Ile397, Ile400, Leu483, and Tyr502. In **19**, the aromatic ring is packed with Leu324, Met365, Val376, Phe378, Phe388, and Phe401. Additionally, the anisole and 3-methylquinoline are surrounded by Leu287, Leu292, Ala327, Val361, Met365, Ala368, and Phe377. Furthermore, the 5-methyl in the isoxazole group contributes to the binding by interacting with Trp317, Cys393, Leu396, and Ile397.

Furthermore, the shape of compounds **9** and **19** matches closely the reciprocal shape of the RORγt binding cavity (Figure 8). However, there are no obvious H-bonding partners for the nitrogen-rich system at the core of **9** and the sulfonamide group of **19** in the predicted binding pose. It is possible that careful modification in these areas could improve their binding with RORγt even further. Amino acids surrounding the nitrogen linker in **9** and the sulfonamide in **19** are hydrophobic, and thus, the introduction of hydrophobic moieties at these positions may be worthwhile.

The notable difference between compounds **9** and **19** is that the chlorophenyl ring in **9** protrudes between the His479–Tyr502 pair and disrupts their H-bonding. The His479 is forced to adopt a different side chain conformation, which is stabilized by the formation of either an H-bond with the ether group of **9** or the backbone carbonyl of Leu475. The disruption of the His479–Tyr502 interaction and loss of the aromatic ring stacking of His479 with Phe506 could decrease the stability of the helix 12 active conformation. However, the energy loss of losing the aromatic ring stacking can be at least partly covered by hydrophobic interactions formed when the His479 packs between Met358 and Leu396. The predicted binding mode of **19** showed a smaller conformational change in contrast to the complete re-orientation of His479 with **9**. Nevertheless, with **19**, the His479 side chain adopted a flipped orientation that was less optimal for the formation of the His479–Tyr502 H-bond (Figure 1).

## 7. Materials and Methods

### 7.1. Protein Structures and Preparation

RORγt structures with a full orthosteric inverse agonist bound were obtained from the Protein Data Bank (PDB; https://www.rcsb.org/; obtained 1 June 2019) [51,52]. The structures were superimposed using VERTAA in the BODIL molecular modeling environment [53], and the validity of side-chain conformations in the ligand-binding site was checked against the electron densities with COOT (version 0.8.9.1) [48]. Two protein 3D structures were selected for VS (PDB IDs: 5NTW [32] and 5VB6 [42]). While 5NTW is a good representative of most inverse agonist bound RORγt structures, 5VB6 presents an exceptional conformation state of the LBD. This unique state is explained by the crystallization process, during which RORγt was covalently tethered to a cofactor peptide stabilizing its structure thermodynamically, which in turn provides higher conformational flexibility. Hence, focusing on 5VB6 may provide new atomic insight into the RORγt inverse agonism [42]. The two structures were prepared with Protein Preparation Wizard in Maestro (Schrödinger Release 2018-2; Schrödinger, LLC, New York, NY, USA, 2018; https://www.schrodinger.com) for molecular docking and NIB modeling. Protonation states were assigned using PROPKA with pH 7.4 ± 0.0, and added hydrogens were optimized with the OPLS3 force field [54]. 

### 7.2. Ligand Structures and Preparation

RORγt inverse agonists used in the NIB model training with BR-NiB were acquired from the ChEMBL database [46] (obtained 10 June 2019; http://dude.docking.org). Only those full orthosteric inverse agonists with reported IC_50_ values of traceable data origin were included. If several activity measurements existed for the same compound, the most potent IC_50_ value was selected. The set comprised 191 active compounds. The SPECS 10 mg compound library, consisting of approximately 170,000 compounds (SPECS, The Netherlands; www.specs.net; obtained 28 June 2019), was used as decoy compounds in VS model optimization (25%) and in actual VS to identify RORγt inverse agonists (100%). This SPECS library was selected for VS due to its suitable size for testing various methods, reasonable pricing, and variability of chemistry. Both ChEMBL and SPECS molecules were processed with LIGPREP (Schrödinger Release 2018-2: LigPrep, Schrödinger, LLC, New York, NY, 2018) to generate possible tautomers and enantiomers with OPLS3 charges [54] at pH 7.4 ± 0.0.

### 7.3. Nib Models

NIB models of the LBD for 5NTW (Model Ia) and 5VB6 (Model IIa) were constructed with PANTHER [5] (version 0.18.19; http://www.medchem.fi/panther/). NIB models were generated as described earlier [55]. Here, the FCC packing method with a filler radius of 0.85 Å and box radius of 20 Å was used. All cavity points that were positioned further than 4.5 Å from the protein were automatically removed. These NIB models served as input models for the greedy search optimization done with BR-NiB (see below).

### 7.4. Molecular Docking and Rescoring

A set of molecular docking and scoring methods were compared to discover the most optimal way of identifying active ligands in VS. The best result was acquired when the ChEMBL compounds were docked with PLANTS [56] (version 1.2; http://www.tcd.uni-konstanz.de/plants_download/) and rescored (--noOptimization) with ShaEP [6] (version 1.3.0) utilizing a NIB model. In PLANTS, the binding site radius was set to 12 Å, and the output docking conformations were clustered with a root-mean-square deviation (RMSD) of 2 Å. The utilized scoring function was ChemPLP, and the search speed was set to speed1. The selected docking and scoring methods did not yield a high correlation coefficient, which can be explained by the heterogeneous nature of the dataset originating from different studies. Consequently, there was broad dispersion among the IC_50_ values of the RORγt inverse agonists as the values typically varied multi-fold for the same compound. Like the ChEMBL compounds, the SPECS compounds were also docked with PLANTS and rescored with ShaEP using the NIB models.

### 7.5. Optimization of Nib Models

The original PANTHER-generated NIB models were optimized using the BR-NiB method [13], ShaEP [6], and ROCKER [15]. The BR-NiB code is available online via GitHub free-of-charge (https://github.com/jvlehtonen/brutenib). In BR-NiB, BEDROC20 [43] was used as the target metric. Randomly selected one-fourth of the SPECS compounds (~50,000) were used as decoy compounds, and all RORγt inverse agonists from ChEMBL (191 compounds) were utilized as active compounds. The optimization was performed for Models Ia and IIa, which, in turn, generated Models Ib and IIb, respectively (Figure 2).

Two additional BR-NiB-optimized NIB models were generated using training sets in which the best-ranked ChEMBL molecules from the first round of optimization were excluded. In other words, those ChEMBL compounds ranked among the best 1% of decoy compounds for Models Ib and IIb were removed. Finally, the original NIB models (Models Ia and IIa) were optimized a second time utilizing the new sets of active compounds yielding Model Ic and Model IIc, respectively (Figure 2).

### 7.6. Pharmacophore Point Filtering

PHA point-based filtering or screening was executed using SDFCONF [49]; (https://sites.utu.fi/sdfconf/) for the top 1% best SPECS compounds pushed forward by each optimized NIB model (Models Ib, c and IIb, c) to obtain a set of potentially active compounds. In SDFCONF, the screening was performed using five important binding regions of the RORγt-LBD (Figure 4). During screening, a compound passed the PHA filter if it overlapped or fitted into regions 1, 2, 3, and 4 or fitted regions 2, 3, 4, and 5. In addition, the compound had to contain a heavy atom within a 4 Å radius from either Tyr502 or His479. The PHA filtering was performed separately for all four groups of SPECS compounds that were ranked among the best 1% based on the ShaEP rescoring. 

### 7.7. Compound Selection

ALogP values were calculated with Canvas [57,58] (Schrödinger Release: 2018-2: Schrödinger, LLC, New York, NY, USA, 2018) for compounds that passed both the BR-NiB and SDFCONF screens. Those compounds with an estimated logP > 5.5 were excluded. The RORγt-LBD is very lipophilic as reflected by the neutrality of NIB models (Figure 2), and thus, lipophilicity may be essential for binding. This observation justified the selection of a relatively high logP threshold value. Potential PAINS compounds were removed with PAINS-filtering (PAINS1-3) in the Canvas program. 

Binding free energy was calculated for the remaining compounds using PRIME [59] MM/GBSA (Schrödinger Release: 2018-2: Prime, Schrödinger, LLC, New York, NY, USA, 2018) to exclude the compounds presenting weak predicted binding energies. The predictions were done individually to the docked compounds in complex with either 5NTW or 5VB6 protein structures. The following options were used: (1) VSGB solvation model and OPLS3 (Optimized Potentials for Liquid Simulations) force field were applied, (2) use input partial charges ON, use implicit membrane OFF, and (3) distance from ligand = 4 Å, sampling method: minimize. The compounds presenting binding energies > −95 were excluded. The remaining molecules were visually evaluated, considering the energy-minimized complexes provided by PRIME MM/GBSA. As a result, after careful visual inspection with preference to core diversity, 28 compounds were selected for experimental testing.

### 7.8. Experimental Testing

The activity of the 28 selected molecules was determined at 5 μM concentration using the Human RAR-related Orphan Receptor Gamma Reporter Assay System in a 96-well format (Indigo Biosciences, State College, PA, USA). The assay utilizes human reporter cells designed to express high levels of human RORγ hybrids of both isoforms 1 and 2 and is capable of quantifying inverse agonism and agonism. The assay was performed as a single data point analysis, and 10 molecules exhibiting the strongest activity were selected for further analysis. The same kit was used for determining the IC_50_ values for the most promising molecules. Concentrations of the tested molecules varied from 10 µM to 8 nM, using 1/3-fold decrements within the range of 6 µM to 8 nM. Otherwise, the kit protocol was followed, and the analysis was done similarly as in a prior study [37]. The IC_50_ value of ursolic acid was determined four times as positive control for the experiments.

### 7.9. Molecular Similarity Analysis

The structural similarity of the in vitro hits with known active compounds for RORγt was analyzed to estimate the structural novelty of the VS hits. All compounds having IC_50_ data for RORγt were downloaded from the ChEMBL database in SMILES format (2587 compounds, obtained 8 February 2023). 64-bit linear molecular fingerprints were generated for all compounds with Canvas in Maestro (Schrödinger Release: 2022-1: Schrödinger, LLC, New York, NY, USA, 2022). Daylight invariant atom types were used. The Tanimoto coefficient, which describes fingerprint overlap, was used as the similarity metric. Completely different or similar molecules get a Tanimoto coefficient of 0 or 1, respectively. Low values of the Tanimoto coefficient (0–0.2) were considered to reflect structural novelty.

### 7.10. Physicochemical and Admet Property Calculations

Physicochemical and ADMET properties were calculated for the active VS hit compounds using QikProp in Maestro (Schrödinger Release: 2018-2, LLC, New York, NY, USA, 2018). The obtained values were evaluated based on the recommended values in QikProp manual. AlogP values were obtained from the aforementioned calculations performed with Canvas.

### 7.11. Figure Preparation

Figure 1, Figure 2 and Figure 8 were prepared using Maestro (Schrödinger Release 2022-3; Schrödinger, LLC, New York, NY, USA, 2018). Figure 3 was prepared using ROCKER [15] (http://www.medchem.fi/rocker). Figure 4 (lower panel) was prepared using PyMOL (version 2.5.0, Schrödinger, LLC). Figure 6 was prepared using GraphPad Prism version 8.4.2 (GraphPad Software, LLC, Boston, MA, USA). The 2D structures in Figure 7 were prepared with 2D Sketcher in Maestro (Schrödinger Release 2018-2; Schrödinger, LLC, New York, NY, USA, USA, 2018).

## 8. Conclusions

This study implemented a novel VS protocol, innovatively combining the use of flexible molecular docking with the cavity-based rescoring methodology BR-NiB, PHA filtering, and MM/GBSA calculations, to discover RORγt inverse agonists. The atomic compositions of the NIB models, which were used at the initial stage in shape/ESP-based docking rescoring, were trimmed using the greedy search method BR-NiB. Previously, this shape-focused PHA modeling technique had only been benchmark tested using multiple drug targets and datasets with random training/test set divisions. By combining the usage of BR-NiB with point-based PHA filtering and MM/GBSA calculations, an effective hit-rate of 29% was achieved with limited experimental testing. In total, eight of the twenty-eight tested small molecules inhibited RORγt at ≤10 μM level. Importantly, compound **19** inhibited the receptor with an IC_50_ value of 1.1 µM. The verified hits represent different structural cores, which show the structural flexibility and diversity of the RORγt inverse agonism and the ability of the introduced VS protocol to facilitate scaffold hopping in docking-based VS campaigning. Overall, this work, along with the previous benchmarking and prospective drug discovery studies, demonstrates that VS protocols built on the NIB methodology can be readily and effectively utilized with various target proteins.

## Figures and Tables

**Table 1:** The experimental results, binding energy predictions, and similarities with known RORγt inverse agonists or antagonists for virtual screening hit compounds.

Compound	Compound Name or SPECS ID	IC_50_ (µM) ^1^	MM/GBSA Energy (kcal/mol)	Max Tanimoto Similarity ^3^
Control	Ursolic acid	0.2	-	-
**1**	AE-641/30156032	Nb	−102.79	-
**2**	AF-399/15599217	NS	−98.34	-
**3**	AF-399/41318865	Nb	−115.72	-
**4**	AF-399/41895766	Nb	−102.31	-
**5**	AF-399/42017292	Nb	−103.83	-
**6**	AF-399/42017398	Nb	−98.77	-
**7**	AF-399/42017933	Nb	−107.31	-
**8**	AG-205/33688028	Nb	−100.63	-
**9**	AG-205/36940103	1.7	−105.53	0.149
**10**	AG-219/11789371	9.7 ^2^	−106.91	0.093
**11**	AG-219/12748006	Nb	−99.60	-
**12**	AG-690/11570086	Nb	−114.9	-
**13**	AG-690/40752975	2.6	−98.67	0.127
**14**	AG-690/40753951	2.3	−99.59	0.121
**15**	AH-487/40936254	7.3	−110.82	0.140
**16**	AH-487/41654264	Nb	−112.1	-
**17**	AK-918/11909161	NS	−102.78	-
**18**	AK-968/12162001	Nb	−93.12	-
**19**	AK-968/15253414	1.1	−97.45	0.171
**20**	AK-968/15359742	Nb	−98.30	-
**21**	AK-968/15607331	10.7 ^2^	−117.16	0.131
**22**	AK-968/15608930	Nb	−99.75	-
**23**	AK-968/41022069	Nb	−104.65	-
**24**	AN-648/15596220	3.8	−110.34	0.462
**25**	AN-989/41838307	2.4	−108.44	0.109
**26**	AN-989/41838397	3.2	−113.62	0.114
**27**	AO-081/15386326	Nb	−107.94	-
**28**	AO-081/15386957	Nb	−103.42	-

^1^ Experimentally determined IC_50_ values. Nb = No detectable binding at 5 µM concentration; NS = Non-soluble. ^2^ Minimum signal was not reached with the measured concentrations in IC_50_ determination. ^3^ Maximum value of Tanimoto coefficient compared to all molecules obtained from the ChEMBL database.

**Table 2:** Calculated physicochemical and ADMET properties of the active virtual screening hit compounds.

Compound	MW ^1^	QPlogKhsa ^2^	% Human Oral Absorption ^3^	HBD ^4^	HBA ^5^	QPPCaco ^6^	AlogP ^7^	RO5 ^8^	#rtvFG ^9^
**9**	509.994	0.297	100	1	10.65	2766.242	4.894	1	0
**13**	547.647	1.63	93.086	1	6.5	991.865	4.795	2	1
**14**	517.621	1.597	90.076	1	5.75	819.461	4.812	2	1
**15**	614.842	0.686	40.411	1	10	4.28	2.823	1	0
**19**	528.581	0.432	78.699	2	10.25	220.29	4.802	1	0
**24**	587.773	0.277	46.497	1	11.7	17.451	1.776	1	1
**25**	538.62	1.131	87.639	3	7	859.654	5.444	2	1
**26**	520.63	1.116	86.241	3	7	804.649	5.239	2	1

^1^ Molecular weight (Da). ^2^ Binding to human serum albumin (Acceptable values: −1.5–1.5). ^3^ >80% high, <25% poor. ^4^ Estimated number of hydrogen bonds donated by the solute to water molecules in an aqueous solution. Values are averages taken over a number of configurations. ^5^ Estimated number of hydrogen bonds accepted by the solute from water molecules in an aqueous solution. Values are averages taken over a number of configurations. ^6^ Predicted Caco-2 cell permeability in nm/sec. <25 poor, >500 great. ^7^ AlogP obtained from Canvas. ^8^ Number of Lipinski’s rule of five violations. ^9^ Number of reactive groups that could cause decomposition, reactivity or toxicity problems in vivo. Recommended value: 0–2.

## Data Availability

The data that support the findings of this study are available from the corresponding author upon reasonable request.

## Abbreviations

ABS	Allosteric binding site
ADME	Absorption, distribution, metabolism, and excretion
BEDROC	Boltzmann-enhanced discrimination of receiver operating characteristic
RMSD	Root-mean-square deviation
R-NiB	Negative image-based rescoring
BR-NiB	Brute force negative image-based
LBR-NiB	Ligand-enhanced brute force negative image-based
LBD	Ligand binding domain
NIB	Negative image-based
PDB	Protein Data Bank
RORγt	Retinoic acid-related orphan receptor γt
VS	Virtual screening
PHA	Pharmacophore
ESP	Electrostatic potential
MM/GBSA	Molecular mechanics/generalized Born surface area

## References

[1] Pagadala N.S., Syed K., Tuszynski J. (2017). Software for molecular docking: A review. Biophys. Rev..

[2] Zoete V., Grosdidier A., Michielin O. (2009). Docking, virtual high throughput screening and in silico fragment-based drug design. J. Cell. Mol. Med..

[3] Kitchen D.B., Decornez H., Furr J.R., Bajorath J. (2004). Docking and scoring in virtual screening for drug discovery: Methods and applications. Nat. Rev. Drug Discov..

[4] Kurkinen S.T., Niinivehmas S., Ahinko M., Lätti S., Pentikäinen O.T., Postila P.A. (2018). Improving docking performance using negative image-based rescoring. Front. Pharmacol..

[5] Niinivehmas S.P., Salokas K., Lätti S., Raunio H., Pentikäinen O.T. (2015). Ultrafast protein structure-based virtual screening with Panther. J. Comput. Aided. Mol. Des..

[6] Vainio M.J., Puranen J.S., Johnson M.S. (2009). ShaEP: Molecular overlay based on shape and electrostatic potential. J. Chem. Inf. Model..

[7] Kurkinen S.T., Lätti S., Pentikäinen O.T., Postila P.A. (2019). Getting Docking into Shape Using Negative Image-Based Rescoring. J. Chem. Inf. Model..

[8] Jokinen E.M., Gopinath K., Kurkinen S.T., Pentikainen O.T. (2021). Detection of binding sites on SARS-CoV-2 Spike protein receptor-binding domain by molecular dynamics simulations in mixed solvents. IEEE/ACM Trans. Comput. Biol. Bioinforma..

[9] Gopinath K., Jokinen E.M., Kurkinen S.T., Pentikäinen O.T. (2020). Screening of Natural Products Targeting SARS-CoV-2–ACE2 Receptor Interface–A MixMD Based HTVS Pipeline. Front. Chem..

[10] Virtanen S.I., Pentikäinen O.T. (2010). Efficient virtual screening using multiple protein conformations described as negative images of the ligand-binding site. J. Chem. Inf. Model..

[11] Niinivehmas S.P.S.P., Virtanen S.I.S.I., Lehtonen J.V.J.V., Postila P.A.P.A., Pentikäinen O.T.O.T. (2011). Comparison of virtual high-throughput screening methods for the identification of phosphodiesterase-5 inhibitors. J. Chem. Inf. Model..

[12] Jokinen E.M., Postila P.A., Ahinko M., Niinivehmas S., Pentikäinen O.T. (2019). Fragment- and negative image-based screening of phosphodiesterase 10A inhibitors. Chem. Biol. Drug Des..

[13] Kurkinen S.T., Lehtonen J.V., Pentikäinen O.T., Postila P.A. (2022). Optimization of Cavity-Based Negative Images to Boost Docking Enrichment in Virtual Screening. J. Chem. Inf. Model..

[14] López-López E., Fernández-de Gortari E., Medina-Franco J.L. (2022). Yes SIR! On the structure–inactivity relationships in drug discovery. Drug Discov. Today.

[15] Lätti S., Niinivehmas S., Pentikäinen O.T. (2016). Rocker: Open source, easy-to-use tool for AUC and enrichment calculations and ROC visualization. J. Cheminform..

[16] Kurkinen S.T., Lehtonen J.V., Pentikäinen O.T., Postila P.A. (2022). Ligand-Enhanced Negative Images Optimized for Docking Rescoring. Int. J. Mol. Sci..

[17] Huang M., Bolin S., Miller H., Ng H.L. (2020). Rorγ structural plasticity and druggability. Int. J. Mol. Sci..

[18] Zhang Y., Luo X.Y., Wu D.H., Xu Y. (2015). ROR nuclear receptors: Structures, related diseases, and drug discovery. Acta Pharmacol. Sin..

[19] Jetten A.M. (2009). Retinoid-related orphan receptors (RORs): Critical roles in development, immunity, circadian rhythm, and cellular metabolism. Nucl. Recept. Signal..

[20] Mickael M.E., Bhaumik S., Basu R. (2020). Retinoid-Related Orphan Receptor RORγt in CD4+ T-Cell–Mediated Intestinal Homeostasis and Inflammation. Am. J. Pathol..

[21] Weawer C.T., Elson C.O., Fouser L.A., Kolls J.K. (2013). The Th17 pathway and inflammatory diseases of the intestines, lungs, and skin. Annu. Rev. Pathol..

[22] Igaki K., Nakamura Y., Komoike Y., Uga K., Shibata A., Ishimura Y., Yamasaki M., Tsukimi Y., Tsuchimori N. (2019). Pharmacological Evaluation of TAK-828F, a Novel Orally Available RORγt Inverse Agonist, on Murine Colitis Model. Inflammation.

[23] Xue X., Soroosh P., De Leon-Tabaldo A., Luna-Roman R., Sablad M., Rozenkrants N., Yu J., Castro G., Banie H., Fung-Leung W.P. (2016). Pharmacologic modulation of RORγt translates to efficacy in preclinical and translational models of psoriasis and inflammatory arthritis. Sci. Rep..

[24] Chang M.R., Lyda B., Kamenecka T.M., Griffin P.R. (2014). Pharmacologic repression of retinoic acid receptor-related orphan nuclear receptor γ is therapeutic in the collagen-induced arthritis experimental model. Arthritis Rheumatol..

[25] Fukase Y., Sato A., Tomata Y., Ochida A., Kono M., Yonemori K., Koga K., Okui T., Yamasaki M., Fujitani Y. (2018). Identification of novel quinazolinedione derivatives as RORγt inverse agonist. Bioorganic Med. Chem..

[26] Kumar N., Lyda B., Chang M.R., Lauer J.L., Solt L.A., Burris T.P., Kamenecka T.M., Griffin P.R. (2012). Identification of SR2211: A potent synthetic RORγ-selective modulator. ACS Chem. Biol..

[27] Sun N., Xie Q., Dang Y., Wang Y. (2021). Agonist Lock Touched and Untouched Retinoic Acid Receptor-Related Orphan Receptor-γt (RORγt) Inverse Agonists: Classification Based on the Molecular Mechanisms of Action. J. Med. Chem..

[28] Jetten A.M., Cook D.N. (2020). (Inverse) Agonists of Retinoic Acid–Related Orphan Receptor γ: Regulation of Immune Responses, Inflammation, and Autoimmune Disease. Annu. Rev. Pharmacol. Toxicol..

[29] Sun N., Guo H., Wang Y. (2019). Retinoic acid receptor-related orphan receptor gamma-t (RORγt) inhibitors in clinical development for the treatment of autoimmune diseases: A patent review (2016-present). Expert Opin. Ther. Pat..

[30] Sun N., Yuan C., Ma X., Wang Y., Gu X., Fu W. (2018). Molecular mechanism of action of RORγt agonists and inverse agonists: Insights from molecular dynamics simulation. Molecules.

[31] Scheepstra M., Leysen S., Van Almen G.C., Miller J.R., Piesvaux J., Kutilek V., Van Eenennaam H., Zhang H., Barr K., Nagpal S. (2015). Identification of an allosteric binding site for RORγt inhibition. Nat. Commun..

[32] Kallen J., Izaac A., Be C., Arista L., Orain D., Kaupmann K., Guntermann C., Hoegenauer K., Hintermann S. (2017). Structural States of RORγt: X-ray Elucidation of Molecular Mechanisms and Binding Interactions for Natural and Synthetic Compounds. ChemMedChem.

[33] Lugar C.W., Clarke C.A., Morphy R., Rudyk H., Sapmaz S., Stites R.E., Vaught G.M., Furness K., Broughton H.B., Durst G.L. (2021). Defining Target Engagement Required for Efficacy in Vivo at the Retinoic Acid Receptor-Related Orphan Receptor C2 (RORγt). J. Med. Chem..

[34] Tan J., Liu H., Huang M., Li N., Tang S., Meng J., Tang S., Zhou H., Kijlstra A., Yang P. (2020). Small molecules targeting RORγt inhibit autoimmune disease by suppressing Th17 cell differentiation. Cell Death Dis..

[35] Song Y., Xue X., Wu X., Wang R., Xing Y., Yan W., Zhou Y., Qian C.N., Zhang Y., Xu Y. (2016). Identification of N -phenyl-2-(N -phenylphenylsulfonamido)acetamides as new RORγ inverse agonists: Virtual screening, structure-based optimization, and biological evaluation. Eur. J. Med. Chem..

[36] Zhang Y., Xue X., Jin X., Song Y., Li J., Luo X., Song M., Yan W., Song H., Xu Y. (2014). Discovery of 2-oxo-1,2-dihydrobenzo[cd]indole-6-sulfonamide derivatives as new RORγ inhibitors using virtual screening, synthesis and biological evaluation. Eur. J. Med. Chem..

[37] Rauhamäki S., Postila P.A., Lätti S., Niinivehmas S., Multamäki E., Liedl K.R., Pentikäinen O.T. (2018). Discovery of Retinoic Acid-Related Orphan Receptor γt Inverse Agonists via Docking and Negative Image-Based Screening. ACS Omega.

[38] Wu Z., Ma H., Liu Z., Zheng L., Yu Z., Cao S., Fang W., Wu L., Li W., Liu G. (2022). wSDTNBI: A novel network-based inference method for virtual screening. Chem. Sci..

[39] Damm-Ganamet K.L., Arora N., Becart S., Edwards J.P., Lebsack A.D., McAllister H.M., Nelen M.I., Rao N.L., Westover L., Wiener J.J.M. (2019). Accelerating Lead Identification by High Throughput Virtual Screening: Prospective Case Studies from the Pharmaceutical Industry. J. Chem. Inf. Model..

[40] Karaś K., Sałkowska A., Walczak-Drzewiecka A., Ryba K., Dastych J., Bachorz R.A., Ratajewski M. (2018). The cardenolides strophanthidin, digoxigenin and dihydroouabain act as activators of the human RORγ/RORγT receptors. Toxicol. Lett..

[41] Li Z., Liu T., He X., Bai C. (2022). The evolution paths of some reprehensive scaffolds of RORγt modulators, a perspective from medicinal chemistry. Eur. J. Med. Chem..

[42] Li X., Anderson M., Collin D., Muegge I., Wan J., Brennan D., Kugler S., Terenzio D., Kennedy C., Lin S. (2017). Structural studies unravel the active conformation of apo RORγt nuclear receptor and a common inverse agonism of two diverse classes of RORγt inhibitors. J. Biol. Chem..

[43] Truchon J.F., Bayly C.I. (2007). Evaluating virtual screening methods: Good and bad metrics for the “early recognition” problem. J. Chem. Inf. Model..

[44] Gaulton A., Bellis L.J., Bento A.P., Chambers J., Davies M., Hersey A., Light Y., McGlinchey S., Michalovich D., Al-Lazikani B. (2012). ChEMBL: A large-scale bioactivity database for drug discovery. Nucleic Acids Res..

[45] Bento A.P., Gaulton A., Hersey A., Bellis L.J., Chambers J., Davies M., Krüger F.A., Light Y., Mak L., McGlinchey S. (2014). The ChEMBL bioactivity database: An update. Nucleic Acids Res..

[46] Gaulton A., Hersey A., Nowotka M., Bento A.P., Chambers J., Mendez D., Mutowo P., Atkinson F., Bellis L.J., Cibrián-Uhalte E. (2017). The ChEMBL database in 2017. Nucleic Acids Res..

[47] Hanley J.A., McNeil B.J. (1982). The meaning and use of the area under a receiver operating characteristic (ROC) curve. Radiology.

[48] Emsley P., Lohkamp B., Scott W.G., Cowtan K. (2010). Features and development of Coot. Acta Crystallogr. Sect. D Biol. Crystallogr..

[49] Lätti S.T., Niinivehmas S., Pentikäinen O.T. (2022). Sdfconf: A Novel, Flexible, and Robust Molecular Data Management Tool. J. Chem. Inf. Model..

[50] Lipinski C.A., Lombardo F., Dominy B.W., Feeney P.J. (2001). Experimental and computational approaches to estimate solubility and permeability in drug discovery and development settings. Adv. Drug Deliv. Rev..

[51] Rose P.W., Prlić A., Altunkaya A., Bi C., Bradley A.R., Christie C.H., Di Costanzo L., Duarte J.M., Dutta S., Feng Z. (2017). The RCSB protein data bank: Integrative view of protein, gene and 3D structural information. Nucleic Acids Res..

[52] Berman H.M., Westbrook J., Feng Z., Gilliland G., Bhat T.N., Weissig H., Shindyalov I.N., Bourne P.E. (2000). The Protein Data Bank. Nucleic Acids Res.

[53] Lehtonen J.V., Still D.J., Rantanen V.V., Ekholm J., Bjorkland D., Iftikhar Z., Huhtala M., Repo S., Jussila A., Jaakkola J. (2004). BODIL: A molecular modeling environment for structure-function analysis and drug design. J. Comput. Aided. Mol. Des..

[54] Harder E., Damm W., Maple J., Wu C., Reboul M., Xiang J.Y., Wang L., Lupyan D., Dahlgren M.K., Knight J.L. (2016). OPLS3: A Force Field Providing Broad Coverage of Drug-like Small Molecules and Proteins. J. Chem. Theory Comput..

[55] Ahinko M., Kurkinen S.T., Niinivehmas S.P., Pentikäinen O.T., Postila P.A. (2019). A Practical Perspective: The Effect of Ligand Conformers on the Negative Image-Based Screening. Int. J. Mol. Sci..

[56] Korb O., Stützle T., Exner T.E. (2006). PLANTS: Application of ant colony optimization to structure-based drug design. Lecture Notes in Computer Science Vol. 4150: Ant Colony Optimization and Swarm Intelligence-ANTS2006 Proceedings, Brussels, Belgium, 4–7 September 2006.

[57] Duan J., Dixon S.L., Lowrie J.F., Sherman W. (2010). Analysis and comparison of 2D fingerprints: Insights into database screening performance using eight fingerprint methods. J. Mol. Graph. Model..

[58] Sastry M., Lowrie J.F., Dixon S.L., Sherman W. (2010). Large-scale systematic analysis of 2D fingerprint methods and parameters to improve virtual screening enrichments. J. Chem. Inf. Model..

[59] Jacobson M.P., Pincus D.L., Rapp C.S., Day T.J.F., Honig B., Shaw D.E., Friesner R.A. (2004). A Hierarchical Approach to All-Atom Protein Loop Prediction. Proteins Struct. Funct. Genet..

